# The COVID-19 paradox of online collaborative education: when you cannot physically meet, you need more social interactions

**DOI:** 10.1016/j.heliyon.2022.e08823

**Published:** 2022-01-24

**Authors:** Eva Kalmar, Tom Aarts, Esther Bosman, Camera Ford, Lisa de Kluijver, Josine Beets, Lisette Veldkamp, Pauline Timmers, Diede Besseling, Joris Koopman, Chuntzu Fan, Enya Berrevoets, Melissa Trotsenburg, Loes Maton, Jill van Remundt, Ela Sari, Lee-Wen Omar, Emiel Beinema, Robbert Winkel, Maarten van der Sanden

**Affiliations:** Communication Design for Innovation, Science Education and Communication, Applied Sciences, TU Delft, the Netherlands

**Keywords:** Online and blended education, COVID-19 outbreak, Higher education, Collaborative learning, Student experience, Collaboration

## Abstract

Collaborative learning is a teaching method that brings together students to discuss a topic important for a given course or curriculum and solve a related problem or create a product. By doing this, learners create knowledge together and gain 21^st^ –century skills such as communication, critical thinking, decision making, leadership and conflict management. Universities had to close their campuses and turn their education fully online in 2020 due to the COVID-19 pandemic, which created a forced step in the evolution of the digitalisation of collaborative teaching. How did TU Delft face this challenge? How did the students experience the online version of collaborative learning? How did distant learning affect their motivation? This article presents four student team projects investigating these questions from the collaborative learning perspective. One of the significant findings of these projects is the lack of socio-emotional interactions during online collaborative work. We present a few guidelines on how to enable these interactions when designing online or blended collaborative education.

## 1 Introduction

The number of people attending university has tremendously increased in the last decades, and digitalisation entered the field of education, resulting in the introduction of distant or remote education methods via digital technologies. Parallel to digitalisation, collaborative and transdisciplinary learning also became an emerging trend in higher education, especially in engineering programs, to train the future experts who can deal with the increasingly complex, multi-stakeholder and multi-perspective nature of the problems our societies face nowadays (Felgueiras et al., 2017). These processes have challenged the classical teaching methods and questioned what has to be taught (Davies, 2009; Chasanidou et al., 2014).

Due to the outbreak of COVID-19 in the Netherlands, the Dutch government chose to suspend on-campus education at all Dutch universities from the 12^th^ of March 2020. Just as other universities around the world, Delft University of Technology (TU Delft) decided to continue the education online: the classical lectures were scheduled to be held on online communication platforms, or lecturers created remote video fragments and posted them on the Virtual Learning Environment. Team discussions were held via video conference tools; practical courses were stopped or partially held online whenever possible.

There have been tremendous studies published since the COVID-19 outbreak on how universities tackled the online shift and what they have learned from this sudden transition (Bao, 2020; Chakraborty et al., 2020; Crawford et al., 2020; Dulamă and Ilovan, 2020; Budur et al., 2021; Doyumgaç et al., 2021). Still, there was little attention so far on the effect of the transitions on teamwork- or design-focused education methods (Wildman et al., 2021). The aim of this article is to discuss how the lockdown in 2020 accidentally brought together and speeded up the trends of digitalisation of university education and collaborative learning, forcing lecturers to teach collaborative and design courses online and to evaluate the effectiveness of this sudden change. We compare the changes the COVID-19 transition generated with the theoretical background synthesised from previous literature about collaborative learning and formulate further suggestions for the short-term future.

In the first part of the article, we give an up-to-date overview of the field of cross-disciplinary and collaborative learning. Later, we summarise the digitalisation of collaborative learning, highlighting this trend's problems and challenges and explaining why this is not a straightforward process. Then we summarise the results of four student groups from the Communication Design for Innovation master track. The teams investigated the effects of the changes TU Delft's lecturers implemented in their collaborative learning courses due to the closure of the campus. The four student research projects answered the following research questions: 1.) How did the sudden offline-online shift affect social interactions and people-place connections? 2.) How did the online transition affect design education? 3.) What are the differences between students' sense of community when using a virtual classroom instead of a traditional one? 4.) What is the link between student motivation and empathy in the context of online education? We present the results relevant from the perspective of collaborative education and discuss these by comparing the findings with the literature. Finally, we formulate recommendations and guidelines for the lecturers, universities and policymakers regarding redesigning teamwork-based courses to online courses and rethinking the concepts and design of already existing online courses to support cross-disciplinary learning better.

## 2 Collaborative and cross-disciplinary learning

Most high-impact scientific research is performed in inter- or transdisciplinary collaborative teams, and the success of these projects depends on the team members ability to work efficiently as a group (Handelsman et al., 2007). Besides scientific research, work done at various governmental or profit-oriented organisations is also shifting towards inter- and transdisciplinarity to tackle multi-stakeholder problems in which the responsibilities are hard to map to a single person, organisation or discipline (Brown et al., 2010). These concerns belong to the category of complex problems. One challenging aspect of these problems is when people try to tackle them from one perspective, the attempt to solve the problem generates further, unforeseen issues for other stakeholders (Brown et al., 2010). Therefore, the complexity of these problems requires the collaboration of multiple groups with conflicting interests and facing multiple worldviews, multiple ways of constructing knowledge, and multiple ethical positions. Unfortunately, this is not what university students generally learn in a disciplinary-focused, subject-based curriculum. To let students experience the process of scientific knowledge creation and transdisciplinary problem solving, we have to make the classrooms similar to communities of professionals by using collaborative learning in university education (Handelsman et al., 2007).

**Collaborative learning** is an instruction method rooted in social constructivism and social learning theories (Bandura and Walters, 1977; Vygotsky, 1980; Wenger, 2011). Its fundamental standpoint is that social discourse is crucial for developing knowledge and cognitive functions (Gerlach, 1994). In collaborative learning, small groups of learners collaborate and communicate with each other to discuss a topic, solve a problem or create a product (MacGregor, 1990). By doing this, they are achieving specific competencies, such as higher-level thinking, critical thinking, oral communication, self-management, leadership, decision making and conflict management skills (Laal and Laal, 2012). In collaborative learning groups, participants are interdependent, and they learn from each other by working together. They help each other, actively collect and share knowledge, and while all of them are held accountable for doing their tasks, they evaluate the team's performance together (Laal and Laal, 2012).

Collaborative learning is the basis for **cross-disciplinary learning**. In a multidisciplinary setup, students come from various disciplinary backgrounds. Social interaction in small cross-disciplinary teams broadens the students' understanding of the perspectives, needs and mindsets of other team members, providing them with the opportunity to learn interdisciplinary or transdisciplinary skills as well. *"In the Collaborative Learning Environment, the learners are challenged both socially and emotionally as they listen to different perspectives, and are required to articulate and defend their ideas*" (Laal and Laal, 2012). In this way, "c*ollective learning is needed to support participants in complex decision-making processes of social change. Solving wicked problems requires mutual learning among the many interested parties, incorporating their multiple ways of thinking* (Brown, 2018)."

Courses based on collaborative learning have different learning materials and structures than individual-based courses, as their focus lies on the interaction between students and lecturers. Students are active knowledge producers through active participation in the collaboration. The collaborative situation created at the beginning of the course is a kind of “social contract” between the peers and the teacher, defining specific conditions under which the interactions may occur (Dillenbourg, 1999). The most significant difference between subject-driven and collaborative learning is that the lecturer is no longer the primary knowledge deliverer but rather the designer of interactive learning (Laal and Laal, 2012). It is the teacher's role to set up the initial conditions of the collaborative environment and monitor and regulate the interactions among students. Instead of providing the correct answer (in the case of complex problems, there are multiple acceptable solutions), the teacher intervenes to redirect the group to a productive direction or solve issues that hinder the collaboration between team members (Dillenbourg, 1999). In return, the high level of interactions between peers and lecturers (Bandura et al., 1999) and the considerable degree of autonomy in the learning process (Lynch and Dembo, 2004) significantly influence student motivation. Moreover, by applying the gained knowledge to practical situations, students experience more relevance and satisfaction (Keller, 2009).

Design education often uses collaborative teaching methods: “*the features of collaboration in design education include effective and efficient communication and reflection, and feasible manipulation of design objects”* (Dreamson, 2017). Especially the social context and collaboration within classical, face-to-face design education are often mentioned as essential aspects of the creative design process. Wragg describes the classical design education program that takes place in a studio as a “*social context in which interactions, feedback and community of practice stem from and extend beyond the studio*” (Wragg, 2019).

In a collaborative setup, learning is happening through specific activities. Some of these activities can be performed individually (like reading, thinking, predicting, etc.), while others can only occur between peers (such as explaining, arguing, questioning, answering, reasoning and co-creating knowledge). These activities trigger unique cognitive mechanisms that only happen in collaborative learning (Dillenbourg, 1999). **Social interactions** that support these cognitive processes are categorised as **cognitive interactions** and can be understood as taking part in two-way communication, responding to turns of talk, providing attention, understanding what is said, planning and reflecting on the teamwork (Isohätälä et al., 2020). To create an environment where people develop effective participatory processes and cognitive interactions, one needs to build trust, commitment, interdependence and shared understanding, and most importantly, embrace failure (Ansell and Gash, 2008). Social interactions that create this emotionally positive environment are categorised as **socio-emotional interactions** (Isohätälä et al., 2020). Teams that show a high level of participation in collaborative learning projects offer high levels of both types of social interactions (Sinha et al., 2015). Multiple studies have shown that social interactions positively influence individual learning through increasing motivation (Bandura et al., 1999). Moreover, students are less likely to quit their studies if peer interactions are common within the study climate (Tinto, 1975; Wilcox et al., 2005; Eggens et al., 2008; Oseguera and Rhee, 2009).

One needs specific **social skills** to be able to create and maintain these social interactions (Johnson et al., 1984). These skills cover seven domains of social behaviour: communication, cooperation, assertion, responsibility, empathy, engagement, and self-control (Little et al., 2017). All of these are important for collaboration and collaborative learning, but these skills rarely stand in the list of learning goals of courses, even if collaborative learning is applied. In this research, we investigate social interactions between students and lecturers, and besides communication and cooperation, we have chosen to focus on the social skill **empathy,** because it was specifically shown to be essential for collaboration amongst students with different backgrounds and cooperation with individuals such as students, lecturers, experts, etc. in a social network in which collaborative education occurs. Contemporary approaches define empathy as a social interaction between individuals wherein one experiences another individual's feelings (Decety and Ickes, 2009). Empathy is a multidimensional construct consisting of a combination of cognitive (perspective taking and fantasy) and emotional (empathic concern and personal distress) concepts (Davis, 1983). **Perspective-taking,** the tendency to spontaneously adopt the psychological point of view of others, was shown to be a key element in creating shared understanding and building positive social interactions (Berenguer, 2007) by generating a sense of psychological closeness between individuals, enhancing social bonds, coordinated interactions and collaborative behaviour (Wald et al., 2016).

In this subchapter, we have introduced cross-disciplinary collaborative learning, a specific instruction method in which student teams explore a complex problem by merging multiple disciplines' concepts, perspectives, and methods. It requires particular course structure and materials to enable the students' cognitive and socio-emotional interactions, which in turn lead to unique learning processes. Students need to learn and master social skills to create and maintain these social interactions. Empathy, especially perspective taking, is one skill that significantly affects building social interactions. Next to empathy, communication and cooperation are central constructs for this research. The next part of the article introduces what is known about the digitalisation of collaborative courses.

## 3 Digitalisation of collaborative education

**Figure 1:**
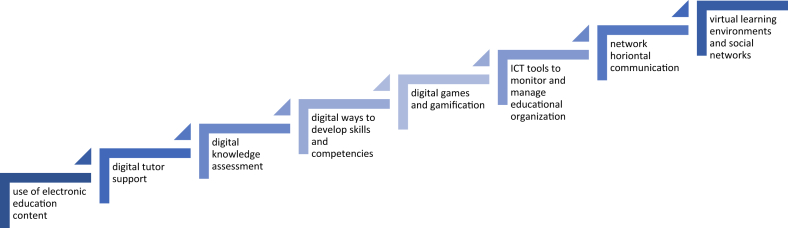
Levels of digitalisation in education, based on Frolova et al., (2020).

The widespread availability of digital learning environments opened new possibilities for universities to deliver knowledge to their students and assess their learning. The digitalisation of education promised a systemic modernisation of the educational space based on digital technologies. In reality, the digitalisation of higher education is happening at various levels (Figure 1) with varied success. The various levels of digitalisation span from the use of digital presentations and video materials during classical lectures to fully online teaching. It covers digital technologies of assessing students' knowledge, online learning environments monitored and managed by the educational organisation, social networks and digital version of serious games and gamification of education (Frolova et al., 2020). In our view, **blended learning**, the approach combining traditional face-to-face elements and online or digital components (Rovai, 2002a, Rovai, 2002b), can be seen as a step in this multilevel perspective.

Online or remote learning is the most prominent and fastest-growing aspect of digitalisation in education. It might sound evident that **online or distance education** is different from classical, face-to-face and blended university education. Still, the aspects in which these two differ might be not that obvious (Keegan, 1988). 1.) In an online setup, teachers and learners are separated throughout the learning process, mostly spatially, but in most cases, separation also occurs in time. Learners can choose to read and watch the course material at any time, can ask questions in an asynchronous communication channel. 2.) The educational organisation influences the planning and preparation of the learning materials and the use of online learning platforms, making institutional online learning different from self-study and private tutoring. 3.) For presenting and distributing educational content, various media technologies are used. 4.) Two-way communication is achieved through a computer network so that students and teachers can perform a dialogue. 5.) The final character, defined by Keegan, is the predominance of individual-based education, with potential group activities for occasional didactic or social purposes (Keegan, 1988).

The best-known examples for online education are Massive Open Online Courses (**MOOCs**). In principle, the teaching methods used in xMOOCs (extended MOOCs), the most widespread form of MOOCs, perfectly fits universities' disciplinary setup to deepen the students' knowledge in a given subject or scientific field. As xMOOCs are adherently designed with the background perspective of individual learning, most current MOOC platforms do not support collaborative or cross-disciplinary learning (Staubitz et al., 2015). Moreover, one of the critical issues online learners often experience is the lack of a sense of community (Rovai, 2002a, Rovai, 2002b).

**Figure 2:**
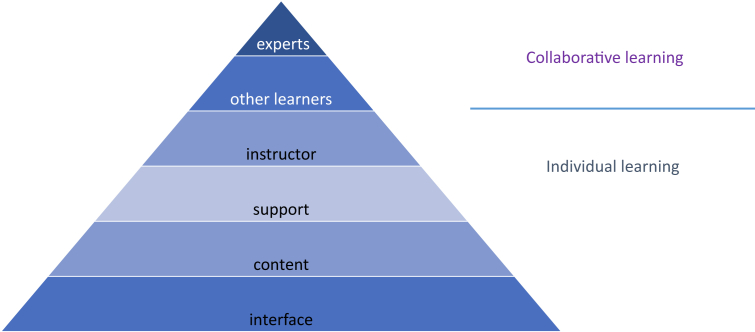
Interactions of learners with other network elements in a collaborative online course (based on Brown, 2018).

To initiate and maintain online social interactions and support cross-disciplinary and collaborative learning, one has to encourage the formation of **learning communities** through collaborative social networks and promote versatile interactions and communications between students as part of the learning (Park and Son, 2010). The learning experience has to be designed in such a way that the learner can interact with 1.) the interface, 2.) the content, 3.) the support, 4.) and the instructor, just like in the case of an xMOOC. Above these, the learner also has to interact with 5.) other learners and 6.) experts or case owners in a real transdisciplinary context (Figure 2) (Brown, 2018).

The social interactions between other learners and experts can create a **sense of community** (McInnerney and Roberts, 2004), which is also essential in determining interactions and participation in online collaborative learning environments (Delahunty et al., 2014). The sense of community is similar to the concept of the sense of presence in virtual environments (IJsselsteijn, 2004), which affects the satisfaction with the interactions within a virtual community (Siriaraya and Siang Ang, 2012). Social presence can be defined as "*the sense of being together and communicating with another person as if he/she was physically present even though he/she is only represented by an avatar and might in fact be miles away*" (Biocca et al., 2001; Felnhofer et al., 2014). **Empathy** was shown to be an essential facilitating factor for social presence in virtual communities (Sas and O'Hare, 2003; Bouchard et al., 2013), highlighting its essential role in online collaborative education. Nicovich and colleagues (2005) claim that "*empathy and presence use the same projective "toolset" to become a part of the experience. Whereas empathy has always been explored as the connection between two individuals […], presence is the connection between individual and environment*” (Nicovich et al., 2005).

One specific type of MOOCs, the connective Massive Open Online Courses (**cMOOCs**) are community-oriented, focusing on creating an online presence. These courses are characterised as: "*based on connection rather than content, which looks more like an online community than a course ... [it] doesn't have a defined curriculum or formal assignments*" (Downes, 2015) cMOOCs often provide their content and promote interaction at the same time through blogs, learning communities and social media platforms (Bali et al., 2015). Next to the cMOOCs, Small Private Online Courses (**SPOCs**) can stimulate collaborative learning by providing social interaction between the learners. These are small-scale courses with a maximum of 20 students (Uijl et al., 2017). Social interactions are part of the course design, for example, by a facilitator assigned to manage learning activities and give personal feedback to learners or an e-moderators who stimulate students to participate actively in the course (Fox, 2013). But even in cMOOCs and SPOCs, in which community-building and social connections are central, only a subset of the human interactions are supported by digital tools, such as virtual meetings, online collaboration spaces, document sharing, and collectively working on documents. These tools belong to the **digitisation of collaborations** and only support the cognitive interactions leaving out than the socio-emotional ones. “*Currently available tools support the three primary aspects of R&D collaboration: communication, work coordination, and knowledge creation and sharing”* (Orellana, 2017). Based on Orellana, this is something different from the **digitalisation of collaborations**. In her view, this latter would mean that the “*subtleties of ideation, problem-solving, and innovation could be captured as digital knowledge—and reused or resold to generate new value’ – via novel digital tools*” (Orellana, 2017).

Based on scientific literature, the current community-focused online courses can support community feeling by digital tools that help students' cognitive social interactions, which are crucial to collaborative learning but fail to support socio-emotional interaction. How about the courses that were given face-to-face but then shifted online? How did TU Delft manage to put its collaborative courses online? Which interactions were supported?

## 4 Digital transition due to COVID-19

TU Delft has formulated in 2016 its guidelines for online education. The Online Learning Experience was created by the Online Learning Course Development Team and holds eight principles. Based on these principles, an online course should be 1) flexible in time, space and content; 2) diverse in activities, resources and assessment; 3) inclusive; 4) supportive in guidance and feedback; 5) interactive; 6) active 7) contextual and finally, 8) innovative (Ribeiro Jorge et al., 2016). Besides these guidelines, at the beginning of the lockdown in 2020, TU Delft lecturers received specific support in the form of a general advice on how to manage the shift. Because many students could have become sick, lecturers were asked to record lectures so that no one would miss essential classes. Brightspace was suggested to be the exclusively used platform to host the online lectures, providing course materials and, in some instances, offering alternatives to proctored exams and a two-way communication channel next to emails. The advice also suggested breaking the teaching into manageable chunks, mentioning that online education does not have to be synchronous or a lecture. Instead, students could watch a video or listen to a podcast, read articles or book chapters covering the given topic, complete assignments with interactive tools, or participate in a discussion in a forum on Brightspace.

The Research Methods in Social Sciences part 1 and 2 courses (SL3111 and SL3531) are part of the Communication Design for Innovation master track of the Science Education and Communication master at TU Delft, teaching social scientific research methods to students coming from different faculties. The students come to this master track with a wide range of experiences and knowledge about social sciences; some have no experience at all, while others have conducted social research before. During these courses, students create a research proposal in a chosen research context that is part of the research portfolio of the CDI team. In eight weeks, they perform the small-scale research in interdisciplinary teams and write up their results in a format of a scientific paper. Collaborative learning stands central in this six-month-long project-based course. The students use the theoretical knowledge gained during the lectures, but they also learn from each other while they discover new theories and concepts that are relevant to their cases and interests. Next to that, they get feedback from their lecturers, case owners and the other teams in the course. In 2020, after the lockdown, the student teams received a new research topic, overarching all the teams. The goal of all the teams was to investigate the effects of the changes implemented due to the lockdown. The different groups picked different aspects of this topic.

Student team 1 investigated the sudden offline-online shift from the perspective of social interactions and people-place connections. They performed exploratory research through qualitative, semi-structured interviews with TU Delft students. The aim of student team 2 was to research the effect of the online transition on design education**.** Qualitative semi-structured interviews were performed via Skype with three students from the Faculty of Architecture and the Faculty of Industrial Design of TU Delft. They were asked about the changes in design education. The results of the qualitative interviews served as the basis for the creation of an online quantitative survey. The goal of student team 3 was to investigate the differences between students’ sense of community when using a virtual classroom instead of the traditional classroom. The team investigated the Research Methods in Social Sciences course itself, in which teamwork and collaborative learning are central. The study focused on social interactions between students and between the lecturer and the students. The research used an already validated classroom community-scale - short form (CCS-SF) instrument (Cho and Demmans Epp, 2019). And finally, student team 4 explored the link between motivation and empathy in the context of online collaborative education via an online survey. It consisted of the interpersonal reactivity index (IRI) (Davis, 1983) to measure empathy and the basic psychological need, satisfaction and frustration scale (BPNSFS) (Chen et al., 2015) to measure motivation. Both questionnaires are validated and widely used.

## 5 Methods

### 5.1 Qualitative, semi-structured interviews about social interaction by team 1

Team 1 focused on how the sudden offline-online shift affected social interactions and people-place connections. The team performed qualitative, semi-structured interviews with ten TU Delft students, representing different nationalities, genders, study backgrounds, progressions within their studies and numbers of years spent studying at TU Delft, recruited via convenience sampling. Two out of the ten students followed an education track that mainly used collaborative learning methods (Architecture and Industrial Design). In total, six students were following courses that included teamwork. To assess their experiences with on-campus and online education, the student team asked questions about the study places and social interactions via online interviews. The interviews were recorded after receiving informed consent from the interviewees, and upon transcription, the recordings were deleted. The semi-structured interview questions were to direct the discussion. The interview questions can be found in Appendix 1. The interviewees' experiences were then analysed through coding, and the differences between on-campus and online education were presented in the following five topics: digital transitions, attitude, study space, collaboration and socialising and free time.

### 5.2 Mixed methods approach by team 2 about the changes in design education

To figure out how the online transition affected design education based on collaborative instruction methods, team 2 performed a qualitative, then a quantitative study. First, semi-structured interviews were performed via Skype with three students from the faculties of Architecture and Industrial Design of TU Delft. The team asked the students about the changes in design education in detail (the interview questions can be found in Appendix 2.). The interviews were transcribed and were subject to subsequent analysis by Atlas Ti. The interviews were coded using the open coding method, where each of the answers given by the interviewees were categorised into one (or multiple) themes. These themes were then divided into the following groups: collaboration, teachers/coaches, media, students, prior situation, course general and others.

To see to what extent were the mentioned issues were valid for a larger population of students, team 2 created an online survey based on the interview results. After introducing the research and asking for signing the informed consent, questions were asked about the different aspects of online communication (which platforms were used, how easy it was to communicate with each other, how efficient was the communication, etc). The detailed questions and the answer options can be found in Appendix 2. The survey was distributed by the student education council of the Architecture Faculty, in total 31 bachelor and master students following collaborative design courses at the Architecture and Industrial Design Faculties filled in all survey questions. Descriptive statistics were performed on the results.

### 5.3 A quantitative questionnaire on community feeling by team 3

To answer the research question “To what extent can the learning environment where the virtual classroom is used sustain the sense of community compared to where the traditional face-to-face classroom is used?”, team 3 investigated the Research Methods in Social Sciences part 1 course. The study covered social interactions between students and between the lecturer and students. The research used a quantitative method to assess the sense of community by using the already validated classroom community-scale - short form (CCS-SF) instrument (Cho and Demmans Epp, 2019). This instrument was originally developed by Rovai (Rovai, 2002a, Rovai, 2002b) and have been revised by follow-up research. The short version, CCS-SF was used in this research, which was tested as reliable and valid as the original form (Cho and Demmans Epp, 2019). This instrument consists of eight self-reported items concerning one of the subscales: connectedness or learning. Each item is a statement indicating the feelings of participating in the course and followed by a five-point Likert scale of potential responses from strongly disagree to strongly agree. The participants selected the answer that was closest to their feelings, and their responses were scored from 1 to 5. See Table 1 for the description of the components of the CCS-SF. The questions of the CCS-SF instrument was put to the online platform of Qualtrics. The survey consisted of three parts: (1) the CCS-SF items for the synchronous online classroom, (2) the CCS-SF items for the physical classroom, and (3) participants' perception about course design, teaching styles and learning styles, and two sections with items for collecting participants’ characteristics and their final comments. As the CCS-SF questions were unaltered, we only present parts 1 and 2 of the survey in Appendix 3. In total, 10 out of the 16 enrolled students filled in the questionnaire. The results of the different learning environments were compared, and non-parametric statistical analysis was applied to compare the mean of the paired samples.

**Table 1:** Connectedness and learning scores that were found worse when using virtual classroom, compared to physical classroom (- means a worse score, = means the same score was and + means that a better score was given to the virtual classroom situation) n = 10 students.

Community aspect	Question in the survey	-	=	+
Connectedness	Q1. I feel that students in this course care about each other	44%	55%	0%
	Q2. I feel connected to others in this course	44%	44%	11%
	Q6. I feel that I can rely on others in this course	0%	100%	0%
	Q8. I feel confident that others will support me	44%	56%	0%
Learning	Q3. I feel that it is hard to get help when I have a question.	44%	44%	11%
	Q4. I feel uneasy when exposing gaps in my understanding	44%	44%	11%
	Q5. I feel reluctant to speak openly	78%	11%	11%
	Q7. I feel that am given ample opportunities to learn	11%	78%	11%

### 5.4 A quantitative online survey by team 4 to measure empathy and motivation of students during online collaborative education

Team 4 formulated two research questions. (1) To what extent does empathy influence students' motivational factors (competence, relatedness and autonomy) to learn through online education? (2) Does the course setup (group project vs studying individually) affect the motivational factors (competence, relatedness and autonomy) of students to learn through online education? A quantitative approach was chosen to quantify the extent of the influence of empathy and course setup in online education by using an online survey. It consisted of the interpersonal reactivity index (IRI) (Davis, 1983) to measure empathy and the basic psychological need, satisfaction and frustration scale (BPNSFS) (Chen et al., 2015) to measure motivation. Both questionnaires are validated and widely used.

A total of 46 completed survey responses were collected. Of the respondents, 59% was female, and 41% were male. 36 out of the 46 followed courses that contained teamwork. As this research aimed to get an idea of the motivational factors of university students overall, this sample of the student population was counted as representative. To calculate the sub-scores of the empathy aspects from the answers received for the IRI part of the online survey, team 4 used the method described by Konrath (2013). This method averages the answer score of all questions per subcategory. Negatively formulated questions were scored in reverse order (5–1 points). The BPNSFS survey measures the motivational factors of respondents in six dimensions: the satisfaction (SF) and frustration (FR) of the three motivational factors: competence, relatedness and autonomy. As with the IRI, the six dimensions are meant to be measured and interpreted as distinct constructs (Cordeiro et al., 2016). Bivariate Pearson correlations were calculated between all possible pairs of sub-scores. The Bayes factor was also computed for all these correlations to determine the statistical significance of the found correlations.

### 5.5 Ethical aspects

The students performing the research were informed about the TU Delft guidelines of Human Research, including the GDPR-regulations. They did not collect personal data and saved their data on TUD-hosted cloud services. They all created informed consent forms for the questionnaires and interviews, in which they explained the goal of their studies, mentioned that participation in the interviews and questionnaires was voluntary, they did not give any gifts to the participants, and the participants could stop answering at any time. All teams filled in the official *Registration Form For Human Research by Students as Part of Regular Courses* form of the Human Research Ethics Committee (HREC) of TU Delft. To qualify for a minimal risk category by this form, students need to avoid collecting confidential or sensitive data, exclude experiments that negatively impact the well-being of participants, not include children and vulnerable people, avoid dangerous situations, and exclude using deception. All student projects were designed in such a way and counted as minimal-risk projects.

## 6 Results

### 6.1 How did the online transition change social interactions? - team 1

The ten semi-structured interviews team 1 performed to figure out how the social interactions and people-place connections were changed after the sudden online transition were analysed by descriptive coding. The results were grouped into five big categories: digital transformations, attitude, study space, collaboration and socialising and free time. In this article, we only present those results that are directly related to collaborative learning.

#### 6.1.1 Digital transitions

The ten interviewees were asked about their opinions on digital interactions compared to communication on-campus. Five students indicated that online conversations were less valuable than physical ones. Students complained about the fatigue from video calls all day, and several students shared their frustrations with the technology. The other reasons online conversations were less effective were related to emotions and subtleties, which were harder to express and interpret in an online setting. One interviewee specifically mentioned that “*it's very different on screen to have emotional interactions”* (I2). Digital technologies made online conversations also harder due to the minor delays caused by weaker internet connections, the weird nature of interrupting others online and the feeling of constantly giving a presentation instead of a discussion.

#### 6.1.2 Study experience and study space

**Figure 3:**
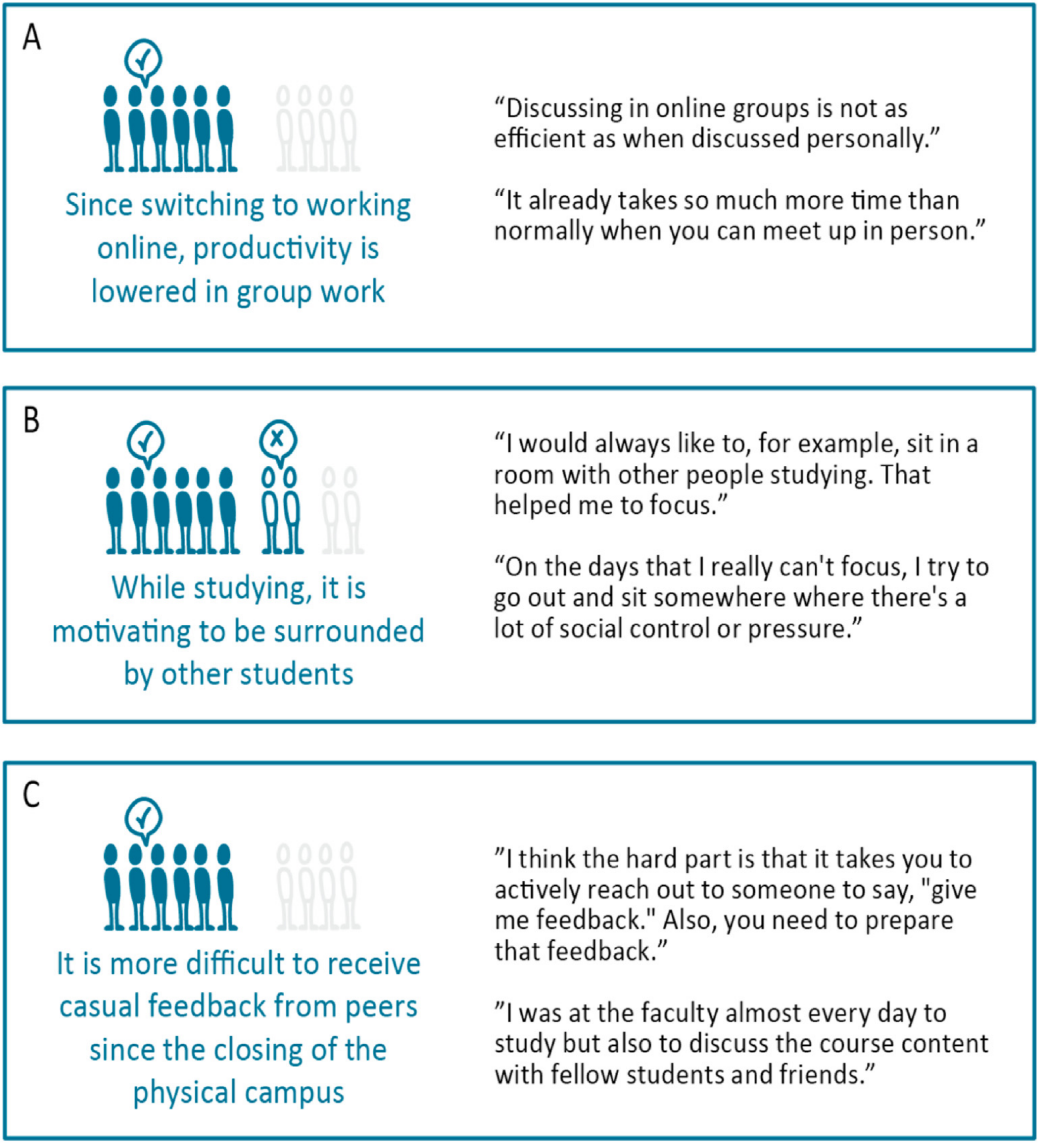
The major differences in social interactions after switching to online education.

Five out of ten interviewees mentioned that the routine of studying was affected by the on-campus - online education change. Besides the routine, one student highlighted that the online switch affected the effectiveness of their learning as well: *“the motivation is equal actually, but somehow I can do less”* (I8). In contrast to this student, seven interviewees mentioned that their home study space lacked certain motivational factors otherwise present at the university. The most prevalent reason for the loss of motivation was the lack of motivation gained from fellow students; all seven students raised this point. *“When I was studying on campus, I would always like to, for example, sit in a room with other people studying. That helped me to focus*” (I3). Besides focusing, studying together had a beneficial effect on the motivation of the six interviewees. *“When you're on campus, and you see other students and you talk with them,[...] they have the same problems. And yeah, it's just really difficult to keep being motivated this way [at home]”* (I2). Four interviewees stated that studying on campus was easier for them because it was a place they associated with learning, while they associated their rooms at home with leisure activities. *“If you were at a place on campus, it would require an extra step, and you can remind yourself 'wait, I'm really here to study, this is a study area, everything around me pushes me to focus on my work'*" (I5). "*It's also not nice that this room is also where I sleep. It's where I play the guitar. It's a room where I do everything, which is kind of annoying. It's less motivating*" (I4). Three interviewees discussed other distractions at home (roommates watching TV or playing loud music) or household tasks like shopping and cleaning that interfered with studying. Some of these results are summarised in Figure 3.

#### 6.1.3 Collaboration

Six out of the ten interviewees mentioned lowered productivity in group work after switching to online education. Two students specifically said that they could not work together as frequently as beforehand, which reduced their motivation and productivity. One student explained it as follows: *“I really get stimulated by group work that's like one of my main motivational factors”* (I5). Another student discussed the negative effect of this drop in motivation the following way: “*In the second part we had to do it from home, couldn't do it physically anymore, and we were just much less motivated. The outcome was much worse.*” (I10). The same interviewee even mentioned meeting with their group despite social distancing rules to complete an otherwise nearly impossible group assignment: “*We realised it's impossible to talk about it over the screen, so we've met up a few times*” (I10).

The transition to distanced group work for two students led to a more individual focus in work. *“It became very decentralised (..). So you say okay, we're with six people, this is a hundred per cent of the work, everybody does one-sixth of the work*” (I6). The other student described discussions before COVID-19 as “*a natural conversation around other things*” and explained that the interactions changed to “*one question you ask on WhatsApp*” (I2). Most of the questions they had were not even asked, as “*it may be quicker to do five minutes of research and figure it out myself”* (I6).

Six of the ten interviewees made clear that there was difficulty obtaining feedback from each other or the lecturer. One of the interviewees even said that some of the feedback was previously obtained unplanned or it came not after asking for it. Compared to that situation, during online education, “*you need to actively reach out to someone to say, give me feedback*.” (I10). Students said they became demotivated by working all by themselves and missing the input from other students. One student specifically mentioned the lack of a benchmark in their work; *“You're missing both the inspiration and the knowledge that you're doing fine and that you're where you should be in your project because you can't look at other people and be like ‘oh, okay. I'm at the same place, or a little behind, or a little forward”* (I10).

Two students tried to replicate the feedback, as received at the faculty, with others in their households. However, due to the great diversity of students living in student houses, there was little overlap in their work. One student explained that as follows: “*Every roommate is doing something else here. So, you can't really motivate each other or complain a little when you don't get something*” (I1).

#### 6.1.4 Socializing and free time

Students also missed the non-learning-related social interactions with friends that typically took place at specific places on campus. The physical presence of a study association provided opportunities to socialise. For some students, even brief, casual in-person contact, such as time spent walking to class or socialising during the lecture break, had been vital because it comprised part of their social time with other students. As a result of the losses of their typical avenues for socialising, one interviewee noted challenges such as feeling alone. Another interviewee mentioned that initiating contact online was difficult, especially with new team members with whom they were less familiar. However, one student did acknowledge that the campus was not necessarily crucial to student socialising: "*It's nice to have a sort of gather point [on campus] where you can see everyone or at least part of your friends. But I don't think you need the campus per se for social interactions*.” Although most of the students appreciated virtual socialising sessions, they also acknowledged that these were less satisfying than their typical in-person social meetings. Some mentioned that it is tiring to call friends via Zoom after a day packed with video calls. Moreover, the dynamics of online socialising were also indicated to be different: “*It's still not very personal. Normally, when you're in a group, you take one person out, and you have a small talk with them about something personal. [It is] really hard in a Zoom session to say hey, let's talk with the two of us*” (I2).

### 6.2 The effect of online shift on design education - team 2

**Figure 4:**
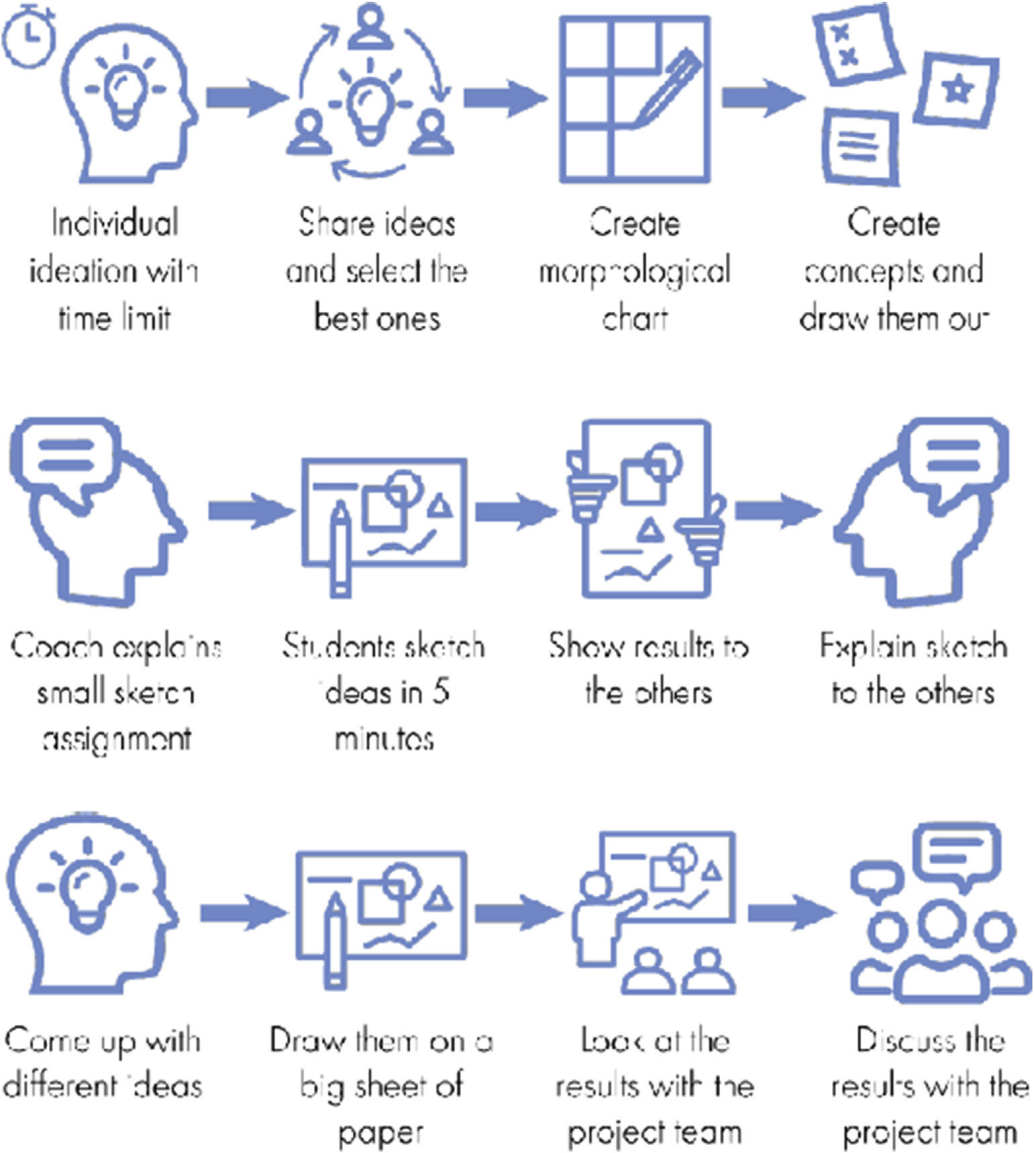
Three types of brainstorm sessions in face-to-face design education.

Team 2 focused on answering the question of how the sudden switch affected the collaboration and creative design processes of students participating in design education. First, they have performed in-depth qualitative interviews. Interviewee W mentioned that drawing and sketching were central to the idea generation sessions in the classical form of design education and that they started the brainstorming process together (Figure 4). They had to put all their ideas into words during the brainstorming sessions instead of sketching them when moving online. That was listed as a major disadvantage. Some of the teams used an online brainstorm platform (Mural, Miro) when they needed to work online, which allowed them to put their ideas on virtual post-its, with the options of moving and re-organising these ideas. Still, these platforms were again used to write down words instead of drawing onto them. “*We were having a Zoom meeting, but then you are talking, and you want to sketch something quickly. That does not work, so you have to put your thoughts into words and make clear to yourself what you mean. Sometimes this takes 5 min, while sketching would have taken you 5 s”* (Interviewee W)

The three students listed several digital tools they were using when they had to move online: such as Microsoft Teams, Skype, WhatsApp or Zoom for online communication and video calls; Miro and Mural for brainstorming; Google Drive and Slack as online storage platforms and finally Adobe Creative Cloud programs for visualisation. They also mentioned issues related to digital communication, such as the lack of non-verbal communication or the lack of spontaneity in their interaction. “*Because communication via Zoom is harder, meetings take unnecessarily long and making decisions is also harder”* (Interviewee W).

Another big change was the decrease in personal contact, decreasing the quality of communication, which had a substantial negative impact on the creative design processes. Interviewee A mentioned that some team members pulled themselves back and did not participate in the teamwork when it turned online. The third student said: *“I think that the difference is that you do everything together [in classical design education]. Now [in online education], I have to completely figure out what to do, I am not sure if it will overlay with shat someone else is doing.”* (Interviewee T)

Team 2 heard stories about fellow students quitting courses because of the difficulties experienced with online teamwork*: “I have the feeling that more people quit courses. Even this week because they think: it is too much.*” (Interviewee T)

Team 2 has generated an online survey covering the points mentioned in the interviews to understand whether other students shared these issues. In total, thirty-one students filled in the questionnaire. Nine of them were following their studies at the Faculty of Architecture and twenty-two at the Faculty of Industrial Design. Six were bachelor students while twenty-five were master students.

**Figure 5:**
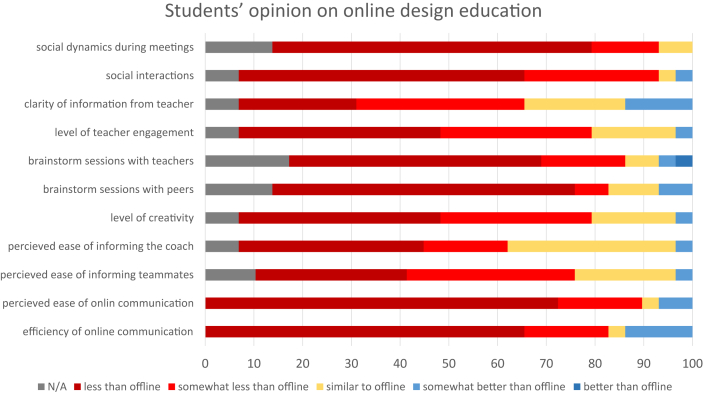
Students' opinion on online design education.

All respondents used at least three digital tools to support their online teamwork, while several students simultaneously used more than six tools. For almost all the questions, most of the students found the online situation worse when compared to the pre-corona case. Roughly three-quarters of the respondents said that the level of creativity (72.4%) and the quality of the brainstorm sessions (69%) were lower when TU Delft moved the design education online. 82.7% of them found that the ways of communication were less efficient in the online setup, and 89.6% found that online communication was harder. 65.5% thought it was more challenging to inform team members online (this was 51% for informing the teacher online), while 20.5% found it as easy as in the classical scenario (it was 34.5% for informing the teacher). 79.3% of the respondents said that the social dynamics during the meetings were less or somewhat less, while 86.2% of them found that the social interactions were less and somewhat less than the offline situation (Figure 5).

### 6.3 Community feeling in face-to-face and virtual classrooms – team 3

Team 3 investigated how the Research Methods in Social Sciences courses 1 was given before and after the lockdown. Ten out of the sixteen enrolled participants filled in the survey. Based on their answers, the comparison of sense of community in a virtual and physical classroom revealed detailed information on which aspects of community feeling changed. For example, almost half of the students indicated that they felt that the fellow students cared less about each other (44%), connected less to others (44%), and supported each other less (44%) in the online setup. In addition, almost half of the students found it harder to get help (44%) or expose gaps in their knowledge in the online versions of the course (44%)s, and more than two-thirds felt that they were more reluctant to speak openly (78%). Surprisingly though, almost all students found that they were given ample opportunities to learn in both versions of the course (summarised in Table 1).

**Figure 6:**
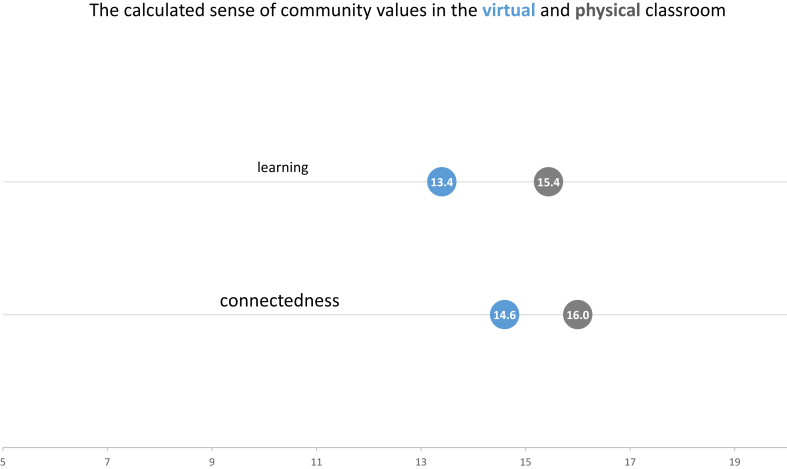
Differences in community scores between the physical and virtual classrooms.

Based on (Cho and Demmans Epp, 2019), the total **learning** score was calculated from the Likert-scale items related to learning. The mean value for learning was 15.4 (SD = 1.2247) for the physical classroom, while 13.4 (SD = 1.81) for the online classroom (Figure 6). The related samples Wilcoxon signed rank test, a non-parametric test to compare the means of paired samples, showed that the difference between the face-to-face and online situation is significant (p = 0.04). The Likert-scale items related to connectedness were added up to form the **connectedness** score. The mean connectedness score was 14.6 (SD = 1.5776) for the online education, while it was 16 (SD = 10.333) for the face-to-face version (Figure 6). The related samples Wilcoxon signed rank test showed the difference between the face-to-face and online situation is significant (p = 0.028). The total **sense of community value** was calculated by summing up the connectedness and learning values. The related samples Wilcoxon signed rank test, showed the difference between the face-to-face (M = 31.4, SD = 2.555) and online situation (M = 28, SD = 2.7487) is significant for the cumulative community value (p = 0.021). These results show that students of the investigated TU Delft course had a **lower sense of community** in the virtual setup.

### 6.4 Motivation and empathy in face-to-face and online cases – team 4

**Figure 7:**
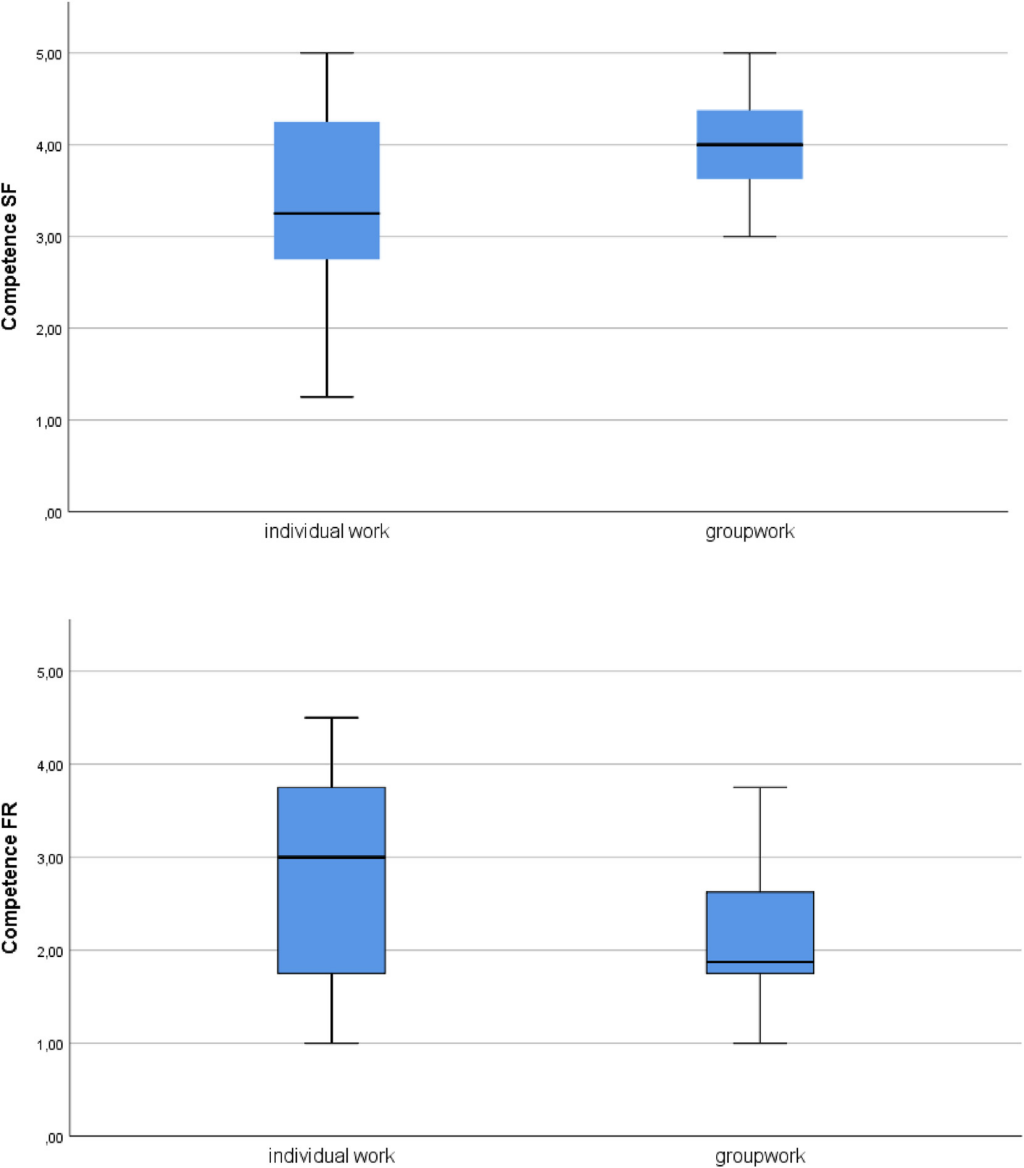
Perceived competence for individual and group work course setup.

Team 4 first investigated whether students following courses based on teamwork had a higher score for different aspects (competence, relatedness and autonomy) of motivation. 18 out of the 46 students (39%) filling in their questionnaire followed a course with group work. Compared to these students, the respondents that focused on a course with individual work had overall lower satisfaction scores in **competence**. As shown in Figure 7, the 18 participants who had a course based on teamwork (M = 3.95, SD = 0.56) demonstrated significantly higher Competence Satisfaction scores, t(44) = -2.7, p = 0.013, compared to the 28 participants who followed a course based on individual work (M = 3.3, SD = 0.9). Additionally, there was a significant effect for Competence Frustration, t(44) = 2.24, p = 0.003 with individual work attaining a higher frustration score (M = 2.8, SD = 1.2) compared to the students having group work (M = 2.1, SD = 0.68).

Next, students’ satisfaction and frustration with regards to **relatedness** (feeling connected) was examined. Again, the satisfaction and frustration score distributions of the two respondent groups were compared. There was no significant effect for relatedness satisfaction and frustration, t(44) = -0.66, p = 0.52 and t(44) = 0.06, p = 0.95. The distribution of **autonomy** satisfaction and frustration for the two groups was also investigated. Although individual work attained lower scores for satisfaction (M = 3.3, SD = 0.8) as well as for frustration (M = 2.9, SD = 0.7) than group work (respectively M = 3.5, SD = 0.6 and M = 3.0, SD = 0.8), there was no significant effect, neither for autonomy satisfaction, t(44) = -0.69, p = 0.49 nor for autonomy frustration, t(44) = -0.52, p = 0.61.

From the data analysis mentioned above, it becomes apparent that there is a significant difference in the motivational factor competence between students that follow courses focusing on individual and group work. Students following courses containing group work had a higher perception of competence. One statement counted for the satisfaction score: *’I feel I can successfully complete difficult tasks.*’ Students doing group work could feel supported by group members, while students with individual work do not have this group to consult or fall back on. A statement that counted for the frustration scale was *’I feel insecure about my abilities*.’

To investigate the role of empathy on motivation in online learning, correlation analysis was performed on the dataset collected by team 4. An overview of all pairwise Pearson's correlation coefficients and corresponding Bayes Factors (BF) between the aspects of empathy and the motivational factors are presented in Table 2. The entries in this table are presented in bold text when the correlation is decisive (BF < 0.01); in regular text when the correlation is strong (0.01 < BF < 0.1) or substantial (0.1 < BF < 0.31); and in italic text when no significant correlation was found (BF > 0.31). This categorisation was taken from Jeffreys (1961), noting that the Bayes factors in Table 2 are inversed with regard to the formulation in this citation.

**Table 2:** Pearson correlations and Bayes factor inference among empathy aspects and motivational factors.

		Empathy: PT	Empathy: EC	Empathy: FS	Empathy: PD	Autonomy SF	Autonomy FR	Relatedness SF	Relatedness FR	Competence SF	Competence FR
Empathy: PT	Pearson Correlation	-									
	Bayes Factor										
Empathy: EC	Pearson Correlation	**0.570**	-								
	Bayes Factor	**0.002**									
Empathy: FS	Pearson Correlation	0.140	0.430	-							
	Bayes Factor	5.790	0.110								
Empathy: PD	Pearson Correlation	0.070	0.230	0.370	-						
	Bayes Factor	7.710	2.560	0.330							
Autonomy SF	Pearson Correlation	**0.530**	0.200	-0.070	-0.260	-					
	Bayes Factor	**0.006**	3.600	7.830	1.940						
Autonomy FR	Pearson Correlation	-0.260	0.040	0.000	0.090	-0.390	-				
	Bayes Factor	2.010	8.380	8.680	7.350	0.223					
Relatedness SF	Pearson Correlation	**0.580**	0.320	-0.010	-0.180	**0.570**	-0.300	-			
	Bayes Factor	**0.001**	0.880	8.640	4.420	**0.002**	1.150				
Relatedness FR	Pearson Correlation	-0.270	-0.140	-0.050	-0.080	-0.130	0.270	-0.330	-		
	Bayes Factor	1.590	5.260	8.270	7.560	5.940	1.670	0.760			
Competence SF	Pearson Correlation	0.290	-0.020	-0.070	-0.360	**0.650**	-0.310	0.370	0.000	-	
	Bayes Factor	1.230	8.590	7.890	0.400	**0.000**	1.030	0.390	6.670		
Competence FR	Pearson Correlation	-0.390	0.030	-0.020	0.170	**-0.570**	0.410	-0.360	0.220	**-0.780**	-
	Bayes Factor	0.260	8.540	8.610	4.600	**0.002**	0.170	0.440	3.040	**0.000**	

As can be seen from Table 2, **perspective taking** was found to be correlated with all of the motivational factors. It showed a substantial relationship with competence frustration, and within the set of empathy aspects, it was also found to have a strong correlation with empathic concern. Most importantly, perspective-taking was found to have strong correlations with both autonomy and relatedness satisfaction. A decisive and robust correlation (Pearson correlation = 0.552; and Bayes factor = 0.003) between perspective taking and the general motivation score was also found.

## 7 Discussion

### 7.1 The shift from campus-based teaching to online education

When TU Delft had to shift its face-to-face education to online teaching, neither the lecturers nor the students were prepared for the consequences of the changes. On the one hand, lecturers had to suddenly become experts in recording videos, navigating digital tools that they had not used before. They had to redesign some of their courses' content, learning goals, materials, and assessment methods to be aligned to these new digital tools. On the other hand, the shift to online education affected students' learning, especially in courses where teamwork is crucial. Using Keegans’ differences between online and offline education introduced in the literature review section (Keegan, 1988), the following trends were identified when online education was introduced at the whole university.

1.) TU Delft, as an educational organisation, steered the planning and preparation of the online learning materials and the use of online learning platforms. A group of experts suggested exclusively using Brightspace as a platform to host the online lectures, providing course materials and, in some instances, offering alternatives to proctored exams, and for a two-way communication channel, next to emails.
2.) The first changes suggested by TUD focused on the transition from synchronous physical activities to digital asynchronous ones, resonating well with the separation of lectures and the lecturers mentioned by Keegan. Lecturers were advised to publish reading and watching materials on Brightspace instead of live, real-time lectures. This made the education materials more available for the students who might become sick or unfamiliar with various digital tools, but lowered the level of interaction and shifted the focus towards individual learning even further. Moreover, due to the shift, students' rooms became lecture and meeting rooms, which were previously associated with fun, leisure time and sleeping. This felt unnatural for some students, as they were used to learning or working together with others on projects mainly in the library or at their faculties. Going to the campus structured their days; being together with other students helped them focus on the work needed to be done, while at home, they experienced various distractions.
3.) During the online education, two-way communication was achieved through emails, Brightspace and various applications, and multiple media technologies were used to present the educational content, as Keegan mentioned. For example, in the case of design education, designer teams used multiple applications for file sharing (Google Drive, Slack, Microsoft Teams), online communication, including video conferencing (Zoom, Skype, Microsoft Teams, WhatsApp or Brightspace's own video tool, the Virtual Classroom), task listing (Trello) or brainstorm-supporting whiteboard (Miro, Mural) applications. Some students used at least six of these different tools in their online collaborations.
4.) The changes mentioned above and the digital tools used during online education mostly supported individual learning. In the case of team projects, these tools provided possibilities to have cognitive social interactions. Students experienced difficulties with online communication. They have found it more tiring; the delays in talking disturbed the normal conversation flow. In addition, they could not talk with one person but with the whole team. Finally, emotions were harder to express and interpret when using online communication channels. Because of these difficulties, socio-emotional interactions were hardly happening via online media.

These trends will be discussed in detail under the subchapters motivation, learning communities, sense of community, and empathy.

### 7.2 Motivation

Based on previous studies, collaborative learning can increase student motivation through the intense interactions between peers and lecturers (Bandura et al., 1999). Student team 4 found that students working together in teamwork-based courses felt more competent than students following individual-focused learning. Competence is an important element of Keller's theory on motivation (Keller, 2008), defining students' expectations and significantly affecting their efforts put in their projects. A student interviewed by team 1 also mentioned being more motivated when learning in teams: *“I really get stimulated by group work that's like one of my main motivational factors”* (I5). Unfortunately, they observed that the shift to online education negatively affected this motivating effect of collaborative work. Six out of the ten interviewees mentioned lowered productivity in group work after switching to online education. Two students specifically mentioned that they were not able to work together as frequently as beforehand lowered their motivation and productivity. However, being together with other students helped them focus on the work that needed to be done.

Some of the experienced difficulties arose because the students could not go to the campus, and their **social interactions** did not happen at the usual place, and the online setup could not support the same type of interactions. Many students missed unplanned at-campus discussions with other students from the same field of study. Social interactions at the university significantly increased university attachment, leading to higher individual motivation to learn (Moghisi et al., 2015). Attachment to a place emerges from the interactions and activities between humans and places and between humans in a particular location (Low and Altman, 1992). Studies on **people-place attachment** (Ross et al., 2010) highlighted the importance of education conducted in a physical place, mentioning that without access to the university, the attachment may decline, which can influence students’ motivation. People-place attachment gives an explanation of how the absence of a physical university campus and physical interactions with fellow students (a community feeling) could cause a negative impact on students' educational experiences with online education during the lockdown.

### 7.3 Learning communities and sense of community

One could potentially compensate for the loss of people-place attachment and encourage online learning communities by setting up collaborative social networks and providing versatile interactions and communications between team members and their coach (Park and Son, 2010). As shown in Figure 2, collaborative online learning should allow a student to interact with the interface, the content, the support, the instructor, but most importantly, with each other and with the case owners or other experts (Brown, 2018).

The tools used to promote collaboration and communication between students during the online education were supporting file sharing, video conferencing, collectively working on documents, synchronous and asynchronous communication to coordinate tasks. Based on Orellana (2017), these digital tools belong to the digitisation of collaborations and only support the **cognitive social interactions** leaving out the socio-emotional ones. Students mentioned that the limited presence of non-verbal communication led to more frequent miscommunication in team meetings. We know from previous studies that social interactions in an online setting are different from the face-to-face situation due to the human-computer interaction standing between human-human interactions. Especially asynchronous communication was shown to make socio-emotional communications more difficult as these are more prone to misinterpretation. Using chats could feel like an unnatural way of socialising compared to coffee-brake discussions (Delahunty et al., 2014). Students could also receive less informal feedback from peers and lecturers during online education; they needed to ask for feedback directly. In general, there were no planned moments for giving feedback online. Students said that because they got tired of participating in video calls all day long, some got frustrated with the technology, so they skipped small talk at the beginning and saw less added value in working together. This is in line with previous studies reporting about students enrolled to online courses showing a significantly higher level of technology-related fear, anger, and helplessness, compared to students taking face-to-face courses (Butz et al., 2015).

The social interactions between other learners and experts can create a **sense of community** (McInnerney and Roberts, 2004), which is also essential in determining interactions and participation in online collaborative learning environments (Delahunty et al., 2014). The student project on the sense of community (team 3) showed that community feeling was lower in the virtual classroom compared to the actual classroom. Some students felt that the other students in the course cared less about each other, felt less connected, and were less confident that the others would support them. This had an effect on their perceptions of learning as well.

The general trend almost all teams could observe is that the lack of informal discussion in teamwork led to **less socio-emotional interaction**, resulting in less motivation of the team members and lowered team productivity. After the lockdown, students taking online design courses felt that they could work online. Still, they were less creative and less effective since they used more verbal communication instead of drawing down their ideas. In addition, they had less personal contact, which had a considerable effect on the quality of their communication. These findings are in line with the previous results showing that online interactions within groups of university students are in general found to be more formal, while informal interactions are the ones that create bonds or strengthen the already existing ones (Haythornthwaite, 2005; Kim and Frick, 2011). In a situation similar to the corona-closure, when students had to transition from face-to-face to online education, social bonds within students' social networks that were already weak before the switch suffered, while bonds already strong before the transition were strengthened by the online switch (Haythornthwaite, 2005). This finding is similar to the so-called Matthew effect (Merton, 1968).

Collaborative learning, as described by Isohätälä and their colleagues, is a process based on social interactions. “*Collaborative learning is a temporally unfolding process, and as such, can only be captured as a series of interactions emerging over time fluctuations of group members’ participation in cognitive and socio-emotional interaction and the characteristics of the moments when concurrent changes in participation and types of interaction occur*” (Isohätälä et al., 2020). They claim that socio-emotional interactions are often not supported even in facilitated teams, although they play a crucial role in the dynamics of the team processes. While cognitive interactions were set up and supported by the lecturers, we also observed that socio-emotional interactions did not get enough attention from the teaching staff. When team members are highly participating in the teamwork, they are generally more engaged in socio-emotional interactions, and when they switch to task-related or cognitive processes, the participation gets lower (Isohätälä et al., 2020). This is not *per se* a problem; this is the natural dynamics of teams. But suppose the online tools do not provide possibilities for sufficient and satisfactory levels of socio-emotional interactions. In that case, the participation of team members in the collaborative work will not increase to the levels needed to sustain their motivation. This can also be behind the fact that students felt they could acquire unexpected and casual feedback more difficultly and that it was harder to get help from their peers when they had a question. The same phenomena can also explain why students felt online teamwork less effective. Their teamwork shifted towards cooperation instead of collaboration, meaning that team members distributed the workload into individual assignments that they added together instead of solving the problem together. The team meetings became less frequent, less effective, and time-consuming, so the students thought they could solve the sub-problems alone much quicker. This turned to be a short-term solution, and in the longer term, they were spiralling down to a much more isolated position.

For some students, the decrease in socio-emotional interaction led to isolation. Unfortunately, due to the lack of a university-wide quantitative study, purely based on our results, we cannot say how severe or widespread this problem is at TU Delft. Still, based on previous and more recent studies (Bartlett, 2008; Huijser et al., 2008; Kwon et al., 2010; Händel et al., 2020), we think this has to be a critical issue to pay attention to.

### 7.4 Empathy

In this research, we investigated social interactions between students and lecturers and have chosen to focus on the social skill **empathy** because it was specifically shown to be essential for collaboration amongst students. **Perspective-taking,** the tendency to spontaneously adopt the psychological point of view of others, was shown to be a key element in creating shared understanding and building positive social interactions (Berenguer, 2007) by generating a sense of psychological closeness between individuals, enhancing social bonds, coordinated interactions and collaborative behaviour (Wald et al., 2016).

Team 4 found a statistically significant correlation between the empathy element perspective-taking and motivation, and in more detail, with three motivation elements: autonomy, relatedness and competence (Deci et al., 1991). Perspective-taking was previously shown to be crucial in building positive social interactions by generating a sense of psychological closeness between individuals, enhancing social bonds, coordinated interactions and collaborative behaviour (Wald et al., 2016), which are concepts related to relatedness. Other studies showed that people with high empathic perspective-taking could easily synchronise their actions to each other in the act of joint music creation (Novembre et al., 2019). This indicates that perspective-taking is important in interpersonal coordination in cooperative teams.

## 8 Moving towards better supported collaborative learning in online education

Our study explored that online collaborative education has a more substantial need for social interaction than we would think. This entails organised contact with other students through teamwork within the course, but overall, lecturers and student counsellors should all make this explicit and therefore discussable. The student teams confirmed the fact described by several academics (Orellana, 2017; Isohätälä et al., 2020), that online education supports mainly the cognitive interactions via the used digital tools, even in courses that are focusing on students working, solving problems, and therefore learning together. At TU Delft, during the first COVID-19 lockdown in 2020, digital tools mainly were used in online collaborative courses to aid document sharing, collective document editing, task-listing, and video conferencing. The cognitive activities supported by these tools were shown to happen in an online environment only when the team members already have a sense of belonging to a meaningful learning community (Rovai, 2002a, Rovai, 2002b). A previous study observed that when students changed from physical to online education, the social interactions in the online setup depended on students’ previous connections. Those social bonds that were already strong before the online switch remained strong, while the weak interactions were further weakened (Haythornthwaite, 2005). This suggests that teams that existed before the lockdown had a higher potential to work in an online setup than newly formed teams because socio-emotional interactions could happen face-to-face in those groups. The reason behind this phenomenon is that developing a new learning community in an online environment takes time and is only accomplished with conscientious effort (Beth et al., 2015). Most course lecturers probably had no previous knowledge on how to create online communities and had no time and existing toolkits to make it happen.

We believe that it is possible to create fully online collaborative courses. For these, one needs to strategically make time and resources available to set up and maintain online communities via planning and facilitating non-task-related socio-emotional interactions. So far, little attention has been spent developing digital tools that support socio-emotional interactions (Jones, 2010), therefore the already existing digital tools should be used for this particular purpose. Universities might require training lecturers of collaborative courses on how socio-emotional interactions could be sufficiently supported with the existing digital tools.

Meanwhile, we believe that **blended education** can provide an intermediate, or in some cases, an ultimate solution, combining online instruction and potentially (formative) assessment with on-campus interaction. In this scenario, the **asynchronous online elements**, the instructions and teaching materials could be available online, before the course where possible, so that students can refer to them at any time. The structure of the online learning environment should allow it to be followed without additional information, with clear expectations and deadlines. If possible, the materials should be personalised, which can increase the feeling of freedom, control, and not lastly, autonomy (He et al., 2020). We suggest incorporating collaborative elements into the online part of the blended learning experience, such as collaborative problem solving, decision making, reflection and case study analysis. These could help students feel less lonely and more competent, increasing their motivation to learn even more. We suggest incorporating teamwork elements or assignments even to those courses that focus mainly on individual learning, with the background notion that teamwork can create a safer environment for students and keep the number of learners high.

Besides the online elements, we suggest planning **physical sessions** when students could meet face-to-face because we found out that it is harder to strengthen the sense of community online (Rovai and Jordan, 2004). We suggest taking transactional distance, social presence, social equality, small group activities, group facilitation, teaching style, learning stage, and community size into account (Rovai, 2002a, Rovai, 2002b) when designing the interactive meetings. Furthermore, we also advise using the concept of people-place attachment, which positively affects motivation and learning. The physical interactions with fellow students at the campus could increase unplanned socio-emotional interactions and peer feedback, but they can also motivate several students to help them learn. Moreover, physical presence in a lecture room can also stimulate the students to ask more questions from the lecturers, strengthening competence.

Finally want to highlight another aspect of creating better online courses based on collaborative learning: teaching and practising social skills. We strongly advise university education programmes to incorporate communication, cooperation, assertion, responsibility, empathy, engagement, and self-control, the social skills required for creating and maintaining effective social interaction in collaborations into their curricula.

## Declarations

### Author contribution statement

Eva Kalmar: Conceived and designed the experiments; Analyzed and interpreted the data; Wrote the paper.

Tom Aarts, Esther Bosman, Camera Ford, Lisa de Kluijver, Josine Beets, Lisette Veldkamp, Pauline Timmers, Diede Besseling, Joris Koopman, Chuntzu Fan, Enya Berrevoets, Melissa Trotsenburg, Loes Maton, Jill van Remundt, Ela Sari, Lee-Wen Omar, Emiel Beinema: Conceived and designed the experiments; Performed the experiments; Analyzed and interpreted the data; Wrote the paper.

Robbert Winkel, Maarten van der Sanden: Contributed reagents, materials, analysis tools or data; Wrote the paper.

### Funding statement

This research did not receive any specific grant from funding agencies in the public, commercial, or not-for-profit sectors.

### Data availability statement

Data will be made available on request.

### Declaration of interests statement

The authors declare no conflict of interest.

### Additional information

No additional information is available for this paper.

## Appendix A Supplementary data

Interview questions Team 1.docx Interview questions Team 1.docx

Interview and survey Questions Team 2.docx Interview and survey Questions Team 2.docx

Survey questions team 3.docx Survey questions team 3.docx
