# Supplementary material for: The COVID-19 paradox of online collaborative education: when you cannot physically meet, you need more social interactions
Source: Heliyon. 2022 Jan 24;8(1):e08823. doi: 10.1016/j.heliyon.2022.e08823 (PMC8810371; doi:10.1016/j.heliyon.2022.e08823)
Supplement: Interview questions Team 1.docx [file mmc1.docx]

Interview questions Team 1

1. Could you tell me a little bit about yourself? Where are you from? What do you study? How long have you been studying at the TU Delft?
2. What is your opinion of your study experience as a TU Delft student in Q1 and Q2 of this year?
   - Was it mostly positive or negative? Why?
   - Were there specific experiences that made this opinion?
   - Has this experience changed since the campus closed? How?
3. How does your home study space differ from where you would study at the TU Delft?
   - Where did you used to study? Why?
   - Where do you study now? Why?
   - How do you feel about these changes or about studying at home? (ask to elaborate)
4. What kind of (social) interactions do you normally have on the physical campus?
   - What are the purposes of these interactions? (could be a study group, lectures, lunch meeting or other social interactions)
   - Can you maintain these (human) interactions in an online setting? If yes, what does that look like?
   - Do these interactions still fulfil their purposes in the same way? If not, what did you try? And why did it fail? How does it make you feel?
5. If you had to pick one place at the TU Delft campus to which you would have access, where everything would be normal, what place would you choose?
   - Why this place?
   - What makes it special?
   - Have you tried recreating it?

To encourage participants to elaborate on their answers, some probing questions were formulated:

- Why do you feel like this?
- What do you think about it?
- How would you do it differently?
- What could be worse?
- What could be better?
- Did it change over time?
