# Supplementary material for: The COVID-19 paradox of online collaborative education: when you cannot physically meet, you need more social interactions
Source: Heliyon. 2022 Jan 24;8(1):e08823. doi: 10.1016/j.heliyon.2022.e08823 (PMC8810371; doi:10.1016/j.heliyon.2022.e08823)
Supplement: Interview and survey Questions Team 2.docx [file mmc2.docx]

Interview Questions Team 2

General questions:

1. What study program/track do you follow?
2. In what year of your study are you currently?
3. Are you a Bachelor or a Master student?

Research specific question:

1. In your current courses, what kind of collaboration is present? Between you and other students and/or your collaboration with teachers?

Research specific questions related to the situation now:

1. How do you collaborate now in the Covid-19 crisis, with fellow students / supervisors / teachers?
   1. To specify this questions: do you work alone or do you collaborate and discuss projects with others students even if you are working on an individual project. Please elaborate on your preferred way of working in the current situation.
2. How much contact do you have with other students during this pandemic?
3. What does this contact look like now that everything is happening online?
   1. Which platforms for communication do you use?
4. If you make any, how do you make prototypes or models now?
5. How is the communication with your teachers/coaches in the current situation?
6. What are the biggest differences/changes you experience during the pandemic, regarding the design education?
7. What were the changes you most struggled with (regarding the collaboration and creative design process), if any?
8. Have you experienced any benefits due to the change to online (design) education?

Research specific questions related to the situation before:

1. How did you collaborate before the Covid-19 crisis, with fellow students / supervisors / teachers?
   1. To specify this question: did you like to work alone or did you collaborate and discuss projects with others students, even if you were working on an individual project. Please elaborate on your preferred way of working.
2. How much contact did you have with other students before the Covid-19 outbreak?
3. What did this contact look like?
   1. Did you (already) use any online media (platforms) to communicate?
4. How did you make prototypes or models before the outbreak? (e.g., physical models, or computer models)
5. How was the communication with your teachers/coaches before the virus outbreak?

Closing questions

1. Are there still themes that we did not discuss, which are important to you?
2. Is there something else you would like to share?

Survey questions Team 2

1. Are you currently taking part in design courses (or have you taken part in design courses) that involve **collaboration**, that were moved online due to the Covid-19 outbreak? Yes / No
2. At which faculty are you currently taking these courses? Architecture and the Built Environment / Industrial Design Engineering / both / other
3. Which level of education are you currently following? Bachelor / Master / PhD / other
4. What **media platform(s)** do you use to collaborate during your design project(s) in the current situation (online education)? *(multiple answers possible)* Adobe Creative Cloud programs / Google Drive / Microsoft Teams / Miro / Mural / Skype / Slack / WhatsApp / Zoom / Other
5. How **efficient** do you find communication in online collaboration, compared to “offline” design education? Better than offline / Somewhat better than offline / Similar to offline / Somewhat less than offline / Less than offline / Don't know / Not applicable
6. How **easy** do you find communication in online collaboration, compared to “offline” design education? Better than offline / Somewhat better than offline / Similar to offline / Somewhat less than offline / Less than offline / Don't know / Not applicable
7. How easy is it to **keep your teammates informed** about your design work, compared to “offline” design education? Better than offline / Somewhat better than offline / Similar to offline / Somewhat less than offline / Less than offline / Don't know / Not applicable
8. How easy is it to **keep your teacher/coaches informed** about your design work, compared to “offline” design education? Better than offline / Somewhat better than offline / Similar to offline / Somewhat less than offline / Less than offline / Don't know / Not applicable
9. What do you think of the **level of creativity** during the design process in online collaboration, compared to “offline” design education? Better than offline / Somewhat better than offline / Similar to offline / Somewhat less than offline / Less than offline / Don't know / Not applicable
10. How would you evaluate the **brainstorm sessions with your teammates,** compared to “offline” design education? Better than offline / Somewhat better than offline / Similar to offline / Somewhat less than offline / Less than offline / Don't know / Not applicable
11. How would you evaluate the **brainstorm sessions with your teacher/coaches**, compared to “offline” design education? Better than offline / Somewhat better than offline / Similar to offline / Somewhat less than offline / Less than offline / Don't know / Not applicable
12. On average, how would you evaluate the **degree of engagement** (e.g., personal contact) of your teacher/coach in online design education, compared to “offline” design education? Better than offline / Somewhat better than offline / Similar to offline / Somewhat less than offline / Less than offline / Don't know / Not applicable
13. On average, how would you evaluate the **clarity of the information** provided by your teacher/coach in online design education, compared to “offline” design education? ? Better than offline / Somewhat better than offline / Similar to offline / Somewhat less than offline / Less than offline / Don't know / Not applicable
14. How would you evaluate the **social interaction** (personal related) during online meetings, compared to “offline” meetings? Better than offline / Somewhat better than offline / Similar to offline / Somewhat less than offline / Less than offline / Don't know / Not applicable
15. How would you evaluate the **social dynamics** (content related) during online meetings, compared to “offline” meetings? Better than offline / Somewhat better than offline / Similar to offline / Somewhat less than offline / Less than offline / Don't know / Not applicable
