# Supplementary material for: The COVID-19 paradox of online collaborative education: when you cannot physically meet, you need more social interactions
Source: Heliyon. 2022 Jan 24;8(1):e08823. doi: 10.1016/j.heliyon.2022.e08823 (PMC8810371; doi:10.1016/j.heliyon.2022.e08823)
Supplement: Survey questions team 3.docx [file mmc3.docx]

**(Basic information)**

1. You are currently: (single choice)

- a graduate student
- an undergraduate student
- other (please specify) (allow text entry)

1. You are currently:

- an enrolled student of this course
- an auditor of this course
- other (please specify) (allow text entry)

1. What is the name of the course you participate in? (text entry)
2. Have you ever participated in the ”synchronous online classroom*” of this course? (single

choice) (force response) *The synchronous and remote, or virtual classroom where

lecturer(s) and students participate through online components, digital tools and the

internet.

- yes
- no

1. Have you ever participated in the ”physical classroom*” of this course? (single choice) (force response)

- yes
- no

1. Have you ever used any asynchronous online learning components given by this course?

(multiple choice)

- yes, using (asynchronous) learning materials offered by the course
- yes, watching the video of recorded synchronous lecture after the lecture
- yes, others (please specify) (allow text entry)
- no

**(Part3)**

**Part 3 Course design, teaching styles and your learning styles**

In the next section, please select the response that comes closest to indicate your perceptions.

23. How do you perceive the overall course design?

- This course has a fixed structure.
- This course is mostly structured with some adjustments occasionally.
- This course has an outline and the contents will be adjusted in accordance with the progress.
- This course is given a subject and the contents are mostly open and flexible.

24. Following the previous question, to what extent do you think the course design is matched

with your learning styles?

- well matched
- somewhat matched
- not quite matched

25. How do you perceive the teaching styles of this course?

- only composed of one-way lecturing
- mainly composed of lecturing with interactions occasionally
- given an online with contents composed of in-course interactions
- mostly composed of dialogue, interactions and discussions

26. Following the previous question, to what extent do you think the teaching styles of this

course are matched with your learning styles?

- well matched
- somewhat matched
- not quite matched

27. How do you define your learning styles?

- mostly active, autonomous
- mostly engaged in learning opportunities but not necessarily active
- mostly passive but attentive when interested
- mostly passive, dependent

**(Last comments)**

Thank you for filling out the survey. Before submitting your responses ...

- Do you have anything else to add on your responses? (text entry)
- Do you have any comments or suggestions on this survey? (text entry)
